# Evaluating Geologic Sources of Arsenic in Well Water in Virginia (USA)

**DOI:** 10.3390/ijerph15040787

**Published:** 2018-04-18

**Authors:** Tiffany VanDerwerker, Lin Zhang, Erin Ling, Brian Benham, Madeline Schreiber

**Affiliations:** 1 Department of Geosciences, Virginia Tech, Blacksburg, VA 24061, USA; tiffanyvanderwerker@gmail.com; 2 Department of Statistics, Virginia Tech, Blacksburg, VA 24061, USA; linzhang@vt.edu; 3 Department of Biological Systems Engineering, Virginia Tech, Blacksburg, VA 24061, USA; ejames@vt.edu (E.L.); benham@vt.edu (B.B.)

**Keywords:** groundwater management, drinking water, water quality, statistical modeling, logistic regression

## Abstract

We investigated if geologic factors are linked to elevated arsenic (As) concentrations above 5 μg/L in well water in the state of Virginia, USA. Using geologic unit data mapped within GIS and two datasets of measured As concentrations in well water (one from public wells, the other from private wells), we evaluated occurrences of elevated As (above 5 μg/L) based on geologic unit. We also constructed a logistic regression model to examine statistical relationships between elevated As and geologic units. Two geologic units, including Triassic-aged sedimentary rocks and Triassic-Jurassic intrusives of the Culpeper Basin in north-central Virginia, had higher occurrences of elevated As in well water than other geologic units in Virginia. Model results support these patterns, showing a higher probability for As occurrence above 5 μg/L in well water in these two units. Due to the lack of observations (<5%) having elevated As concentrations in our data set, our model cannot be used to predict As concentrations in other parts of the state. However, our results are useful for identifying areas of Virginia, defined by underlying geology, that are more likely to have elevated As concentrations in well water. Due to the ease of obtaining publicly available data and the accessibility of GIS, this study approach can be applied to other areas with existing datasets of As concentrations in well water and accessible data on geology.

## 1. Introduction

Worldwide, it is estimated that at least 140 million people drink well water with unsafe concentrations of arsenic (As) [1]. Elevated As concentrations in groundwater occur naturally in many countries [2], with examples in China [3,4], Southeast Asia [5,6,7], Latin America [8,9], Africa [10,11], the United States [12,13,14], and Canada [15,16]. Since As is a known toxin and carcinogen [17,18], drinking water standards for As have been established by the World Health Organization (WHO), the European Union (EU), and the U.S. Environmental Protection Agency (USEPA), among other agencies. Currently, the drinking water standard for As set by the WHO, EU, and USEPA is 10 μg/L, although many studies indicate that adverse health effects may be caused by ingesting As concentrations lower than 10 μg/L [19,20,21]. Such adverse health effects include heart disease [19,22], complications during pregnancy and quality of life in exposed children [23], and diabetes [24,25].

Arsenic is a component of over 200 naturally occurring minerals including sulfides, oxides, and silicates [26]. In addition, As can adsorb to mineral surfaces, including metal oxides and clays [27]. Release of As from these naturally occurring minerals into groundwater can result from mineral dissolution and/or desorption of As from the mineral surface; release can occur under in situ conditions, but can be exacerbated by human activities such as mining. For example, oxidation of As-bearing sulfides has also been linked with elevated As concentrations in groundwater [28,29,30,31]. Arsenic release to groundwater can also occur through desorption via changes in pH or concentrations of competitive anions, such as phosphate [32,33], or changes in As speciation, such as reductive desorption [26]. The mechanism that has resulted in the most widespread release of As to groundwater is reductive dissolution of naturally occurring As-bearing iron oxides, coupled with oxidation of organic matter [1,34,35,36]. Recent studies in the Coastal Plain of Maryland and New Jersey suggest that reductive dissolution of As-bearing glauconite may also be responsible for As release to groundwater [37,38,39].

In addition to naturally occurring minerals, there are many anthropogenic sources of As that can locally impact As concentrations in groundwater, including the use of arsenical herbicides and pesticides [40,41,42], animal feed additives [43,44,45], wood preservation [46,47], mining activities [48,49,50], waste sites [13,51,52], and other sources including smelter operations, combustion of fossil-fuels, some types of glass production, and disposal of bullets, mildew resistant paints, and lead batteries [26,53].

Because As is odorless, colorless, and tasteless, it is difficult for human senses to detect As in water. The primary method for identifying As in well water is through sample collection and analysis. Although public water supplies in the U.S. are regulated under the Safe Drinking Water Act, private wells are not regulated, leaving homeowners with the responsibility for having their wells tested. In addition, well sampling and analysis can be expensive; thus, many homeowners do not regularly sample and test their wells, if at all [54,55,56,57]. Even when wells are found to have elevated As, it is often difficult to determine which source/sources is/are responsible, creating a challenge for regional-scale groundwater protection. Thus, other approaches for evaluating As risk are needed. 

Statistical modeling is one approach that has been used to identify areas susceptible to As contamination and factors that are associated with elevated As in groundwater. Logistic regression, for example, can predict the probability of binary outcomes (e.g., As concentration > 5 μg/L (*Y* = 1) vs. As concentration ≤ 5 μg/L (*Y* = 0) in groundwater) and can also quantify the importance of different variables, such as geologic formation, soil series, and groundwater chemistry associated with As concentrations. Results of previous studies have demonstrated the utility of logistic regression methods for evaluating the relative importance of geological and environmental factors influencing As in groundwater at regional scales and for predicting As concentrations in groundwater in regions where no sampling data are available [6,14,58,59,60,61,62,63,64].

Although national surveys of As concentrations in groundwater supplies have been conducted in the U.S., e.g., [13], fewer than 10% of counties in mid-Atlantic states have been included in these surveys [63]. Evaluation of As in groundwater in Virginia, in particular, has never before been conducted, likely because Virginia has not been identified as an As “hot spot.” However, a recent study [65] identified elevated As within sedimentary aquifers of the Mesozoic Basins of the eastern U.S. and in metamorphosed clastic sedimentary units of the Piedmont and Blue Ridge aquifers, both of which are present in Virginia. There are 2900 wells in Virginia used for public water supply [66]. In addition, approximately 1.6 million Virginia residents use private wells [66]; with a current (March 2018) population of 8.5 million, an estimated 20% of the population uses private well water. Thus, delineating regions with groundwater susceptible to elevated As is a public health concern. The objectives of this study are to evaluate the presence of As in well water in Virginia, to examine the spatial distribution of As concentrations in well water, and to develop a logistic regression model to evaluate if elevated As occurrences are associated with specific geologic units. 

## 2. Materials and Methods

### 2.1. Arsenic Concentrations in Well Water

For this study, we used state-wide datasets from the Virginia Department of Health (VDH) and the Virginia Household Water Quality Program (VAHWQP; www.wellwater.bse.vt.edu), a Virginia Cooperative Extension program based at Virginia Tech. We also searched for groundwater quality data from the Virginia Department of Environmental Quality (VADEQ) and the U.S. Geological Survey (USGS), but datasets available from these agencies contained few samples that were analyzed for As or had other issues such as not retaining reporting limits. Chapman et al. [65] present data on 94 samples in Virginia and had low reporting limits (1 μg/L), but the data represent filtered samples. The datasets we used from VDH and VAHWQP include data for unfiltered samples (see more details below).

Datasets were first checked for duplicate samples. If more than one sample was collected at an individual location, the maximum As concentration for each location was retained and the remainder of the samples were discarded, a method that has been used in similar studies [14,62,67,68] to allow for preservation of as many “events” (i.e., As concentration > 5 μg/L) and minimize small-sample bias in the model results. Summary information about the datasets is included in Table 1.

The VDH dataset contains As concentrations from unfiltered samples collected from public water supply wells in Virginia from 1973 to 2013. The original dataset did not include latitude and longitude for the well locations but did include a general location description for each well, which was used to assign spatial location using Google Earth satellite imagery. If locations could not be not clearly identified, the data were discarded. Samples were collected at the wellhead prior to treatment. As a general guideline, wells were pumped for ~15 min prior to sampling. Reporting limits for As measurements were included in the dataset, but the analytical method used to measure As was not. Thirteen samples had a reporting limit exceeding 5 μg/L (As ranging from 6 to 50 μg/L) and were removed from the dataset. 

The VAHWQP dataset contains concentrations of As (and other water quality parameters) in water samples collected from wells, springs, and cisterns by homeowners. VAHWQP conducts county-based drinking water clinics across Virginia. Samples were not filtered prior to analysis. Arsenic was analyzed in samples collected from 2008 to 2015. The reporting limit for As samples in this dataset is 1 μg/L. Two samples were collected at each location: a first draw sample and a flushed sample. The first draw sample was collected after stagnation in the plumbing (typically overnight). The flushed sample was collected after water was flushed through pipes for at least 5 min. Data from flushed samples were used in this study, as they likely represent a more accurate depiction of groundwater chemistry with less influence from household plumbing. Homeowner-submitted samples were analyzed and results returned confidentially. In addition to collecting the water sample, homeowners completed a survey that documents the water source (e.g., well, spring, or cistern). We removed samples that were collected from springs and cisterns and only kept samples collected from wells. Homeowners were also asked other questions, including perceived condition of the water (e.g., color, odor, taste), information on water treatment systems, and proximity to perceived potential sources of contamination. We did not remove any samples based on homeowner-supplied information on water treatment, as we were not able to check this information for accuracy.

### 2.2. GIS Data

Location of each sample in the datasets was mapped spatially in ArcGIS version 10.2.2 in separate project files [69]. Geologic unit layers were added to each project file to represent environmental attributes. The geologic unit layer (Figure S1) was obtained from the USGS website (https://mrdata.usgs.gov/geology/state/state.php?state=VA) as a shapefile. The shapefile includes bedrock geologic unit name, spatial locations, and a short description. These geologic units (160) were classified first by age, then stratigraphy. Stratigraphic units are only used where they illustrate a special geologic feature and where the age of the units is uncertain. In general, most of the geologic systems that form outcrops can be separated into several comprehensive time-stratigraphic units. In the eastern U.S., hybrid nomenclature is used to describe units that form outcrop bands too narrow to be separated, or that the two units form a homogeneous body of rocks. 

Other spatial data, including land use, lithology, physiographic province, and soils, were also mapped in GIS and were included in our early modeling efforts but were not used in the final modeling. Initially, we were particularly interested in land use because some human activities, including abandoned mines, landfills, toxic waste sites, golf courses, and historical fruit orchards, may be sources of As. However, upon closer examination of the land use data (see [70] for more information), we recognized that the land use categories do not include the specific land uses that would be relevant for As and thus, we changed our focus to evaluating geologic sources. 

### 2.3. Model Creation and Variable Selection

The logistic regression model was built to measure the probability that As concentrations exceed a given threshold:

(1) $$P\left(Y=1|X_{1},\;\ldots ,\;X_{k}\right)=\frac{e^{\beta_{0}+\beta_{1}X_{1}+\beta_{2}X_{2}+\ldots +\beta_{k}X_{k}}}{1+e^{\beta_{0}+\beta_{1}X_{1}+\beta_{2}X_{2}+\ldots +\beta_{k}X_{k}}}$$

where *P*(*Y* = 1|*X*_1_, …, *X_k_*) = is the probability that *Y* = 1 occurred; when *Y* = 1, a sample has an As concentration greater than the threshold; when *Y* = 0, a sample has an As concentration less than or equal to the threshold. *X*_1_, *X*_2_, …, *X_k_* are the regressors (discussed below), and *β*_1_, *β*_2_, …, *β_k_* are the coefficients. The data were analyzed using the statistical software R [71]. We utilized Least Absolute Shrinkage and Selection Operator (LASSO) logistic regression [72] to conduct variable selection and ridge logistic regression [73] to fit the data to obtain robust inference. Both LASSO and ridge logistic regressions are penalized logistic regressions. Compared with regular logistic regressions, penalized logistic regressions include extra regularization terms in the loss function. The loss function is then minimized to get the estimation of the regression coefficients. The regularization term for LASSO is the sum of absolute values of the regression coefficients (L1 penalty), and the regularization term for ridge is the sum of squared values of the regression coefficients (L2 penalty). LASSO can push the estimated regression coefficients to zero, thus it can be used to conduct variable selection, while ridge can stabilize the variance of the estimated regression coefficients in the presence of multicollinearity. Bootstrap analysis was conducted in the variable selection step (LASSO logistic regression). If a candidate variable (geologic unit) was selected (i.e., non-zero) more than 80% of times among 1000 bootstrap samples, this variable was considered as “significant” and was included in the model-fitting step. We calculated the means and 95% confidence intervals of the coefficients of these significant variables based on 1000 bootstrap samples. In the model-fitting step (i.e., ridge logistic regression), significant regressors were identified using a p-value less than 0.01. 

Regressors considered during model selection included the 160 geologic units found in Virginia. These regressors are categorical, which means they have a fixed number of possible values that do not indicate rank or order. These categorical variables were then coded as binary variables or “indicator variables” for each level, following the strategy used by Ayotte et al. [60].

The final model was constructed using the combined VDH and VAHWQP datasets. In the combined dataset, 98.3% of the 5632 observations used in the model were ≤5 μg/L (threshold for model). Other studies that have one dataset separate the data into two sections (e.g., 85% of data and 15% of data) in order to train and validate the model, respectively (see [60] for an example). However, due to the low number of samples above the reporting limit (5.9%) and above the threshold (1.7%) in our dataset, using a combined dataset allowed for a more powerful model.

We used 5 μg/L as the threshold to construct our binary response variable because the reporting limits for the datasets were 1 μg/L (VAHWQP) and 5 μg/L (VDH); thus, using a threshold of 5 μg/L allowed us to use concentrations from both datasets. Using a threshold that was higher than the reporting limit (e.g., 10 μg/L, which is the USEPA’s drinking water standard) was tested during model development, but this introduced higher uncertainty, as we had few samples with As concentrations above 10 μg/L.

We evaluated model performance using several methods. False positive rates and false negative rates were computed upon completion of the regression model. Fitted probabilities of elevated As occurrences from the model were obtained, and the probabilities greater than 0.5 were determined to be As concentrations above the threshold (5 μg/L), that is *Y* = 1, and probabilities less than 0.5 were determined to be As concentrations less than the threshold (5 μg/L), that is *Y* = 0. True positives, true negatives, false positives, and false negatives were then counted and model performance measures (e.g., false positive rate, false negative rate, sensitivity, and specificity) were computed. We also used the Hosmer-Lemeshow goodness-of-fit test, the mean squared error (MSE), and Pearson residuals to compare the observed to fitted values for the model. 

## 3. Results

### 3.1. Spatial Distribution of As in Well Water in Virginia

**Figure 1:**
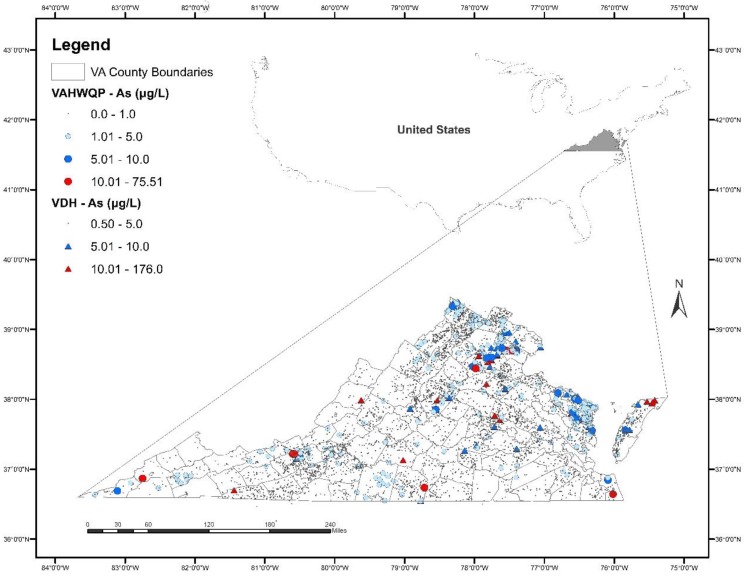
Distribution of arsenic (As) concentrations in well water in Virginia based on the Virginia Department of Health (VDH) and Virginia Household Water Quality Program (VAHWQP) datasets. Outline of the U.S. showing Virginia in the inset map.

Overall, As concentrations are low in well water in Virginia. The spatial distribution of As concentrations in the VDH and VAHWQP datasets is shown in Figure 1 and the concentration distribution is shown in Table 2. The minimum As concentration is <1 μg/L; the maximum As concentration is 176 μg/L. The majority of samples (95% in the VDH dataset; 99% in the VAHWQP dataset) contained As concentrations ≤5 μg/L. Only a small percentage of samples (2.7% in VDH; 0.52% in VAHWQP) had concentrations between 5 and 10 μg/L; similar percentages (2.3% in VDH; 0.23% in VAHWQP) of samples had As concentrations > 10 μg/L. 

The distribution of As concentrations in well water in different geologic units is shown in Table 3. To help evaluate connections between the spatial distribution of As in well water with geologic units, we calculated the percent of samples that exceeded 5 μg/L As (the threshold) for each geologic unit. Units with exceedances above 15% include Tr (Triassic sedimentary rocks) and Tri (Triassic-Jurassic intrusives), and S (Silurian shales and limestones). Units with exceedances between 10% and 15% include lK (lower Cretaceous metamorphic rocks), Pzmi (Paleozoic mafic intrusives, and Tm (Tertiary gravels and sands). Units with exceedances between 5% and 10% include Pzg2 (middle Paleozoic granitic and metamorphic rocks), D (Devonian shales and sandstones), DS (Devonian and Silurian shales and limestones), Tx (Paleocene sands and gravels), Qp (Pleistocene sands and gravels), Z (sedimentary and metamorphic rocks), Mm4 (granitic gneiss), and Ce (Cambrian metamorphic and volcanic rocks).

We also examined the number of samples that exceed 5 µg/L As for different physiographic regions of Virginia (Table 4). Overall, the highest percent exceedance for As in well water is within the Appalachian Plateau, but this province is undersampled (*n* = 14). The Coastal Plain and the Piedmont each have 2–2.5% of samples exceeding the threshold. The Blue Ridge and Valley and Ridge provinces have the lowest percent of exceedances (0.3% and 1.0%, respectively).

### 3.2. Logistic Regression Modeling Results

Results of the variable selection using LASSO logistic regression and the VAHWQP dataset are shown in Table 5. The significant geologic units were identified with an absolute value of coefficient greater than 0. Although p-values cannot be computed using the current LASSO logistic regression function in R, we used the bootstrap analysis to calculate confidence intervals for the coefficients. 

Results of the final model are summarized in Table 6. In the final model, two geologic units were identified as having a higher probability of elevated well water As occurrences: Triassic-aged sedimentary rocks (Tr) and Triassic-Jurassic aged intrusives (Tri). 

**Figure 2:**
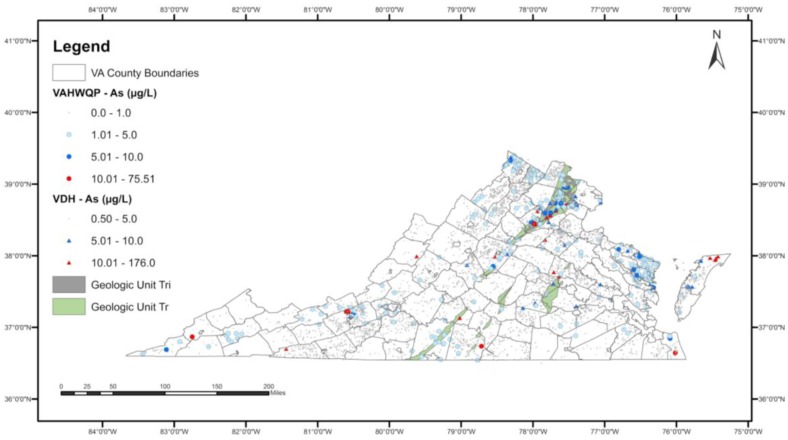
Spatial extent of significant geologic units (Tr: Triassic sedimentary rocks; Tri: Triassic-Jurassic intrusives) in the final logistic regression model overlaid on the spatial distribution of As concentrations.

Figure 2 shows the spatial extent of the two geologic units (Triassic-aged sedimentary rocks—Tr and Triassic-Jurassic intrusives—Tri) that have a higher probability of observing elevated As in well water overlaid on the As concentrations data. 

The equation for the ridge logistic regression model (tuning parameter *λ* = 0.002) with significant (*p* < 0.01) regressors is:

(2) $$Logit\;\left(y\right)=-4.3559+2.2621x_{1}+1.8032x_{2}$$

where $Logit=\frac{P\left(y=1\right)}{1-P\left(y=1\right)}$; *x*_1_ is geologic unit Tri and *x*_2_ is geologic unit Tr.

Results from the evaluation of model fit analysis (Table 7) show that although the model had high accuracy (98%), due to the fact that “negative observations” (As concentrations below the threshold of 5 μg/L) dominate the dataset (98.4% of data are below the threshold), the model cannot correctly predict “positive observations” (i.e., true positives equal 0). The p-value associated with the Hosmer-Lemeshow goodness-of-fit test (2.2 × 10^−16^) suggests the overall model fit was poor, likely a result of the low number of samples above the 5 μg/L threshold. However, the mean squared error of the final model was 0.0556 (closer to zero is better) and the Pearson residuals are generally between −2 and 0. Despite the inability of the model to accurately predict elevated As concentrations in well water in areas where data do not exist, the model is still useful for evaluating the geologic sources of As in well water, which was the primary goal of this study.

## 4. Discussion

### 4.1. Significant Geologic Units

#### Triassic-Aged Sedimentary Rocks and Triassic-Jurassic Intrusives

In the Triassic sedimentary rocks (Tr) and the Triassic-Jurassic intrusives (Tri), between 15% and 23% of samples exceed As concentrations of 5 μg/L, respectively (Table 3). These high percentages of exceedances support results of the logistic regression model, which show that presence of Triassic-aged sedimentary rocks (Tr) has a 6.0-fold (where 6.069 is e^β^; Table 6) greater chance of having elevated As concentrations (>5 μg/L) than when the unit is not present. Similarly, the presence of the Triassic-Jurassic intrusives (Tri) has a 13-fold (where 12.963 is e^β^; Table 6) greater chance of having elevated As concentrations than when this unit is not present. 

**Figure 3:**
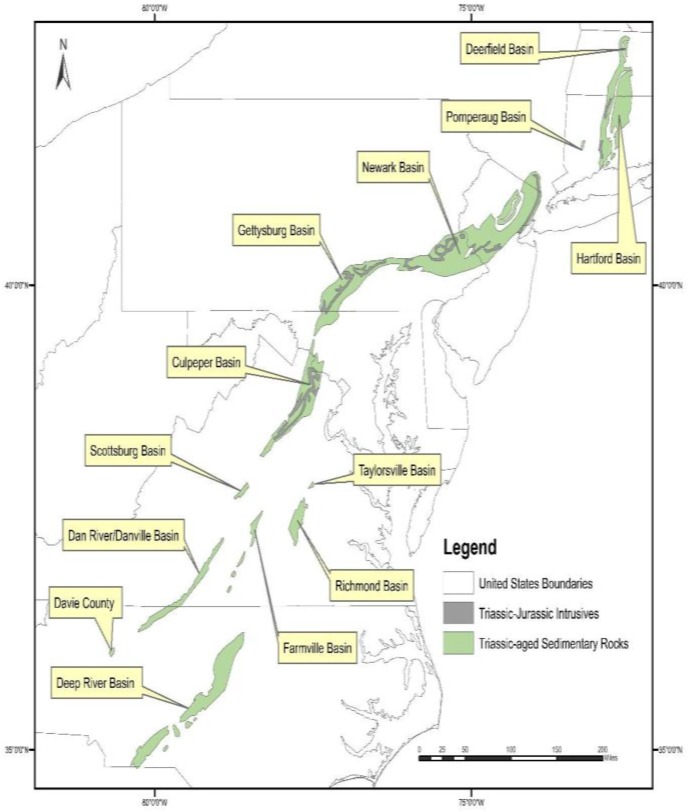
Location of the Mesozoic rift basin complex along the east coast of the U.S. GIS data obtained from the U.S. Geological Survey.

**Figure 4:**
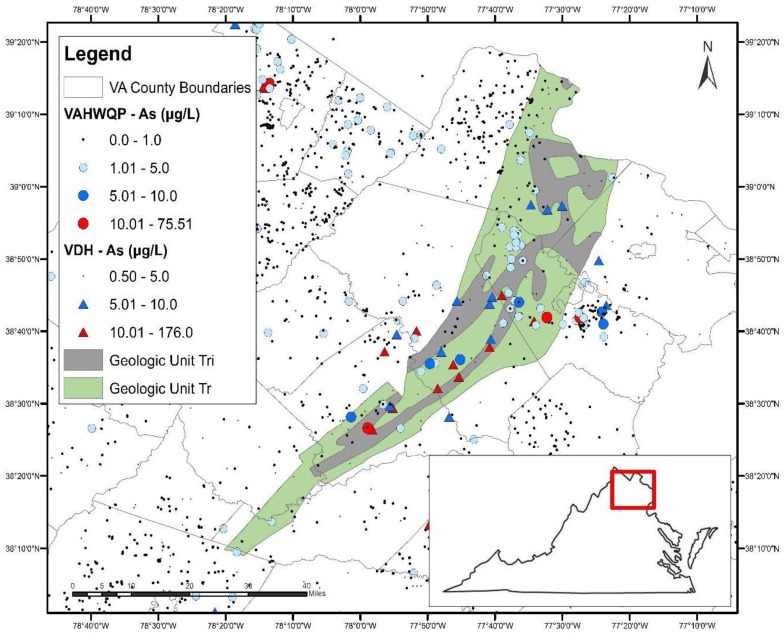
The Culpeper Basin located in north-central Virginia (inset) with overlays of Triassic-aged sedimentary rocks (green), the Triassic-Jurassic intrusives (grey) and As concentrations in well water.

Both of these units are part of the Culpeper Basin within the Mesozoic rift basin complex (Figure 3), which extends from North Carolina to Connecticut. The spatial extent of the Triassic sedimentary rocks and the Triassic-Jurassic intrusives in the Culpeper Basin, overlaid on As concentrations in well water, is shown in Figure 4.

Previous studies have documented elevated As concentrations in groundwater in the Mesozoic rift complex, associated with clastic lacustrine rocks and metamorphosed sedimentary rocks within the Newark and Gettysburg basins (see Figure 3) [28,65,74,75,76]. In the Newark Basin, source rocks for elevated As in groundwater have been identified as the Lockatong formation, a black and grey shale deposited in a lacustrine setting and the Passaic Formation, a red mudstone/siltstone, deposited in a playa [74,77]. Research conducted on the Newark Basin [28,74,78,79] suggests that As-bearing pyrite in black/gray shales of the Lockatong Formation is the primary source of As. In contrast, As mobilization from the red mudstones/siltstone (Passaic Formation) is thought to be triggered by desorption reactions from iron/manganese oxides and clays. Regression modeling [62] shows that high predicted probabilities of elevated As in groundwater in Pennsylvania correspond to high groundwater pH, supporting a pH dependent desorption mechanism. 

Although previous studies have not specifically addressed As concentrations in groundwater in the Culpeper Basin, information gathered from other basins within the rift complex can be applied, as the Newark, Gettysburg, and Culpeper Basins are thought to have been connected during sedimentation and, therefore, sediments within these three basins are likely very similar [80,81,82]. The Lockatong and Passaic formations of the Newark basin generally correlate with the Manassas Formation and Balls Bluff Siltstone, respectively, found in the Culpeper Basin [80].

It is important to note that there are other basins in Virginia associated with the Mesozoic rift complex (see Figure 3), including the Scottsburg, Danville, Taylorsville, Richmond, and Farmville basins. Because our dataset did not include many wells in these other basins, we are not able to assess if these other basins have elevated As in well water. 

### 4.2. Other Regions of Interest

#### 4.2.1. Devonian shales and Sandstones

Although unit D (Devonian shales and sandstones) was not identified in the final model as being significant, a cluster of samples with elevated As concentrations from both datasets occurs in northwestern Virginia in Frederick County (see Figure 1). This area is underlain by the Devonian Hampshire Formation, composed of terrestrial brown and green sandy shales with thin bedded sandstones and red beds [83]. This unit is of interest because the Maryland Geological Survey found that approximately 20% of groundwater samples collected from wells in the Hampshire Formation in Garrett County, Maryland contained As concentrations greater than 10 μg/L [84]. In comparison, As exceedances were <8% for other geologic formations in the county. Additional sampling of wells in this unit is recommended. 

#### 4.2.2. Coastal Plain

Our combined dataset shows elevated As concentrations in 29 wells in the Coastal Plain (Table 4). Although variable selection identified two geologic units of the Coastal Plain (Tm, Qp) as significant variables, the final logistic regression model did not identify either of these units to be significant with respect to As in well water. This is likely due to the fact that the Coastal Plain is a multilayered aquifer system, as well as other factors, such as geochemical conditions, that likely influence As release to groundwater.

Although a previous study on groundwater quality in the Coastal Plain of Virginia did not identify As as an element of concern [83], other studies have documented elevated As concentrations in specific Coastal Plain aquifers of Maryland and New Jersey [37,38,39,85,86,87], prompting us to investigate further. 

Since neither of our datasets included well depth or any information about the aquifer, we were not able to explore statistical relationships between As concentrations, well depth, and aquifer type. However, in collaboration with the VADEQ and the USGS, we found information on a well-by-well basis on screened intervals combined with the top and bottom elevations of Coastal Plain aquifers and confining units to identify to which aquifer each well was open. Five wells with elevated As concentrations in the Northern Neck region of the Coastal Plain were investigated where two were open to the Piney Point aquifer, and one each was open to the Aquia, Potomac, and Yorktown-Eastover Aquifers. For wells in the Eastern Shore, we were unable to get specific information on well depths; however, these wells are likely open to the Yorktown-Eastover Aquifer, as the deeper aquifers contain saltwater and are thus nonpotable [88]. 

Results of this analysis show that the few incidences of elevated As concentrations in well water from our datasets are not associated with any specific aquifer but are found in both shallow (Yorktown-Eastover) and deeper (Piney Point, Aquia, and Potomac) aquifers. Similar results were found by [83], in which As was detected in almost every aquifer and confining unit in the Virginia Coastal Plain, but with the exception of a few samples, concentrations were low (177 samples; maximum 24 μg/L mean 1.8 μg/L, median 1.0 μg/L). Another study [89] involving regional groundwater quality in the surficial aquifer (Yorktown-Eastover Aquifer in VA; Pocomoke Aquifer in MD) of the Eastern Shore found that approximately 50% of wells sampled in the surficial aquifer had As > 0.1 μg/L; however, with a few exceptions, concentrations were below 10 μg/L. 

The reasons underlying the differences in patterns of groundwater As between the Coastal Plain aquifers of New Jersey and Maryland with those of Virginia are currently unclear. The thickness and spatial extent of confining units, the presence of As-bearing minerals such as glauconite, groundwater chemistry (including pH), the presence of competing anions like phosphate, and the availability of dissolved organic carbon that can drive reductive processes that can mobilize As from glauconite and other Fe rich minerals likely all play a role. Due to the lack of information about well construction and groundwater chemistry, we are not currently able to address this and recommend further work to answer this question.

### 4.3. Study Limitations

In this study, we chose to combine two well water datasets, one collected from public water supply wells by a state agency and the other from private wells by homeowners, with each having different collection methods, time spans, and analytical methods, among other important differences, to allow broader spatial coverage across the state. We realize the limitations of combining these data. However, despite these differences, we observe that the spatial data patterns from the two datasets generally support each other; in areas where the VAHWQP samples show elevated As, the VDH samples show similar patterns (see Figure 1). Even with the combined dataset, there are areas of Virginia that have poor spatial distribution of samples, including counties in western, far southwestern, and southern Virginia. Well testing in these areas with poor spatial distribution of samples is recommended.

Second, we focused specifically on geologic sources of As to groundwater. We realize that human sources and activities may also release As to the environment; however, our land use dataset did not include specific land uses that would be relevant to As (e.g., mining sites, landfills, historic orchards, and specific industries where As is or was involved) to allow us to examine non-geologic sources using the logistic regression model. 

Third, our dataset does not include information on well construction (e.g., well depth, screened interval), which limits our ability to identify exactly to what unit the well is open. This made the evaluation of As in the Coastal Plain particularly challenging, as the Coastal Plain is underlain by a multilayered aquifer system. 

Last, an important limitation for the logistic regression model is the issue of small sample-size for the “positive observations” (As > 5 μg/L). Although this is good news for Virginia’s well water quality, the small number of samples exceeding the threshold of the model makes it unusable for prediction. Future work will include developing logistic regression models for regions identified by this study as having geologic units with higher probability of elevated As in well water. Since these smaller regions have more “positive observations”, we should be able to avoid the problem of small-sample bias and develop a more robust model for prediction.

## 5. Conclusions

Arsenic concentrations in well water in Virginia are generally low; only 1.7% of 5632 samples examined for this study exceed As concentrations of 5 μg/L. Logistic regression modeling suggests that these elevated As concentrations are associated with specific geologic units: Triassic-aged sedimentary rocks and Triassic-Jurassic intrusives of the Culpeper Basin in north-central Virginia. While the model developed for this study was successful for evaluating potential geologic sources of As to well water in Virginia, the poor model fit, which results from few samples in our dataset that exceed the threshold value of 5 μg/L, indicates that it should not be used for prediction. However, with drinking water quality surveys such as this one, the purpose is not necessarily to develop a predictive model, but to identify areas where wells should be tested for As or other toxic elements of concerns. These results can be used to help state agencies identify areas of concern for well water quality and to encourage homeowners in these areas of concern to have their wells tested. 

## Figures and Tables

**Table 1:** Summary of datasets used for this study. VDH = Virginia Department of Health; VAHWQP = Virginia Household Water Quality Program.

Dataset Variable	VDH Dataset	VAHWQP Dataset
Number of samples in dataset	10,261	6739
Number of duplicates removed	9043	2325
Final number of samples	1218	4414
Arsenic Reporting Limit (RL)	5 μg/L	1 μg/L
Sample collection dates	1973–2013	2008–2015
Filtration	None	None
Number of samples below RL	1157	4144
Number of samples above RL	61	270

**Table 2:** Concentration ranges for each dataset and their respective percentages.

Concentration Range (μg/L)	VDH Dataset	VAHWQP Dataset
≤5.00	95%	99.25%
5.01–10.00	2.7%	0.52%
>10.00	2.3%	0.23%

**Table 3:** Distribution of As concentrations from VDH and VAHWQP datasets for geologic units in Virginia.

Geologic Unit	*n* > 10 μg/L	5 < *n* < 10 μg/L	*n* < 5 μg/L	Total *n*	% *n* > 5 μg/L
C—Cambrian shales and limestones	0	11	569	580	2
Ce—Cambrian metamorphic/volcanic	3	15	369	387	5
Cq—lower Cambrian clastic rocks	0	0	83	83	0
Cv—Cambrian volcanic rocks	0	6	192	198	3
D—Devonian-aged shales/sandstones	1	9	115	125	8
DS—Devonian-Silurian shales and limestones	1	1	39	41	5
lK—lower Cretaceous metamorphic rocks	0	5	31	36	14
M—Mississippian dolostone and sandstone	2	0	52	54	4
Mm1—felsic paragneiss and schist	0	9	303	312	3
Mm4—granite gneiss	1	7	157	165	5
O—Ordovician shales and dolostones	0	7	504	511	1
Oe—Ordovician metamorphic rocks	0	0	30	30	0
PP1—Atokan and Morrowan Series	0	0	18	18	0
Pzg1—lower Paleozoic granitic/metamorphic	0	5	118	123	4
Pzg2—middle Paleozoic granitic/metamorphic	1	4	48	53	9
Pzmi—Paleozoic mafic intrusives	0	3	27	30	10
Qp—Pleistocene sands	5	15	321	341	6
S—Silurian shales and limestones	3	9	57	69	17
Te—Eocene sands and gravels	0	2	48	50	4
Tm—Tertiary gravels and sands	3	78	746	827	10
Tr—Triassic sedimentary rocks	7	24	180	211	15
Tri—Triassic-Jurassic intrusives	4	10	46	60	23
Tx—Paleocene sands and gravels	0	3	37	40	8
Um—ultramafic rocks	0	0	6	6	0
Ya—Anorthosite	0	0	3	3	0
Ygn—Proterozoic volcanic/metamorphic rocks	0	12	572	584	2
Ym—Paragneiss and schist	0	0	6	6	0
Z—sedimentary and metamorphic rocks	2	22	441	465	5
Zg—granitic and metamorphic rocks	0	0	10	10	0
Zv—volcanic rocks	2	6	206	214	4

**Table 4:** Total number of samples, number of samples that exceed 5 µg/L As, and percent of samples that exceed 5 µg/L from the combined dataset, separated by physiographic province in Virginia.

Physiographic Province	*n*	*n* >5 µg/L	% *n* > 5 µg/L
Coastal Plain	1162	29	2.5
Piedmont	2211	47	2.1
Blue Ridge	749	2	0.3
Valley and Ridge	1496	15	1.0
Appalachian Plateau	14	1	7.1
**Total**	**5632**	**94**	**1.7**

**Table 5:** Results of variable selection.

Geologic Unit	Coefficient (Mean)	95% Confidence Interval, Lower Bound	95% Confidence Interval, Upper Bound
C—Cambrian shales and limestones	−0.779	−2.220	−0.027
Ce—Cambrian metamorphic and volcanic rocks	0.817	0.050	1.714
Cq—lower Cambrian clastic rocks	−0.668	−1.693	−0.100
D—Devonian shales/sandstones	1.060	−0.659	2.317
DS—Devonian and Silurian shales and limestones	0.777	−1.106	2.578
lK—lower Cretaceous metamorphic rocks	0.854	−0.998	2.668
M—Mississippian dolostone and sandstone	0.485	−1.174	2.280
O—Ordovician shales and dolostones	−1.633	−2.664	−0.905
Pzg2—middle Paleozoic granitic/metamorphic rocks	0.420	−1.266	2.275
Pzmi—Paleozoic mafic intrusives	1.065	−0.989	2.750
Qp—Pleistocene sands	1.151	0.240	2.130
S—Silurian shales and limestones	1.490	−0.510	2.841
Te—Eocene sands and gravels	−0.527	−1.529	−0.039
Tm—Tertiary gravels and sands	0.891	0.137	1.822
Tr—Triassic sedimentary rocks	2.054	1.155	3.018
Tri—Triassic-Jurassic intrusives	2.745	1.368	3.875
Tx—Paleocene sands and gravels	0.781	−1.065	2.625
Ygn—Proterozoic volcanic and metamorphic rocks	−1.694	−2.669	−0.971

**Table 6:** Results of the logistic regression model using both datasets (λ = 0.002, using cross validation). Positive coefficients reflect increased probability of As occurrence.

Geologic Unit	Coefficient (*β*)	Exp (*β*)	Standard Error	*T*-Statistic	*p*-Value
Tri—Triassic-Jurassic intrusives	2.5621	12.963	0.4602	5.567	0.0000
Tr—Triassic sedimentary rocks	1.8032	6.069	0.275	5.507	0.0000

**Table 7:** Classification functions for model. TP = True Positive; P = Positive Instances; TN = True Negative; N = Negative Instances; FP = False Positive and; FN = False Negative. NA= not applicable (TP and FP are = 0).

Classification Function	Formula	Value
True Positive Rate (Sensitivity)	TP/P	0%
True Negative Rate (Specificity)	TN/N	100%
Positive Predictive Value (Precision)	TP/(TP + FP)	NA
Negative Predictive Value	TN/(TN + FN)	94.99%
False Positive Rate	FP/N	0%
False Negative Rate	FN/(TP + FN)	100%
Accuracy	(TP + TN)/(TP + FN + FP + TN)	97.87%

## Supplementary Materials

The following are available online at http://www.mdpi.com/1660-4601/15/4/787/s1, Figure S1: Spatial extent of geologic units in Virginia based on age. Data obtained from the U.S. Geological Survey.
