# Supplementary material for: Evaluating Geologic Sources of Arsenic in Well Water in Virginia (USA)
Source: Int J Environ Res Public Health. 2018 Apr 18;15(4):787. doi: 10.3390/ijerph15040787 (PMC5923829; doi:10.3390/ijerph15040787)
Supplement: Supplementary file 1 [file ijerph-15-00787-s001.pdf]

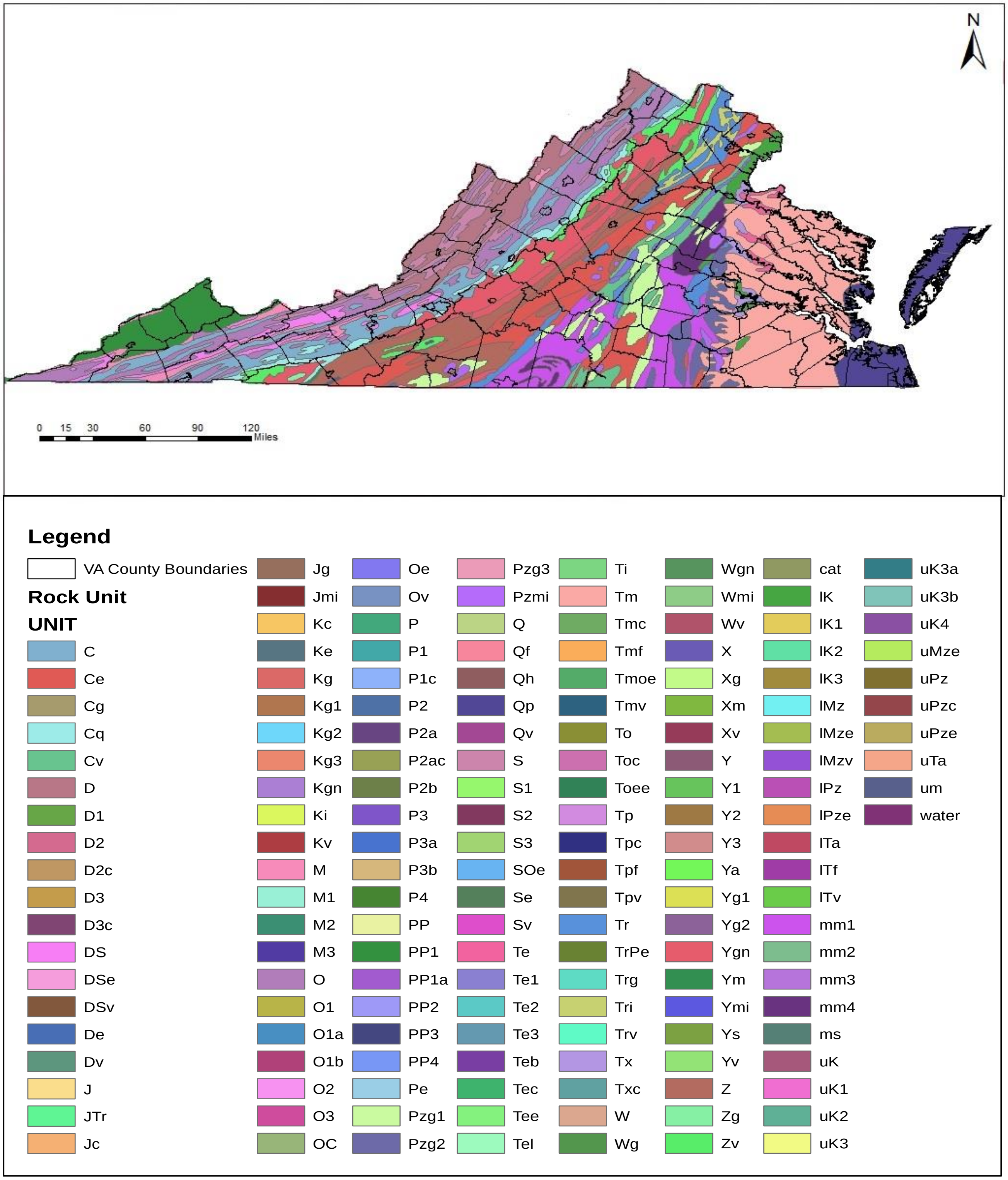

Figure S1. Spatial extent of geologic units in Virginia based on age. Data obtained from the U.S. Geological Survey.
